# Pre-exposure to non-pathogenic bacteria does not protect *Drosophila* against the entomopathogenic bacterium *Photorhabdus*

**DOI:** 10.1371/journal.pone.0205256

**Published:** 2018-10-31

**Authors:** Jelena Patrnogic, Julio Cesar Castillo, Upasana Shokal, Shruti Yadav, Eric Kenney, Christa Heryanto, Yaprak Ozakman, Ioannis Eleftherianos

**Affiliations:** 1 Infection and Innate Immunity Lab, Department of Biological Sciences, George Washington University, Washington, District of Columbia, United States of America; 2 Laboratory of Malaria and Vector Research, National Institute of Allergy and Infectious Diseases, National Institutes of Health, Rockville, Maryland, United States of America; Biomedical Sciences Research Center Alexander Fleming, GREECE

## Abstract

Immune priming in insects involves an initial challenge with a non-pathogenic microbe or exposure to a low dose of pathogenic microorganisms, which provides a certain degree of protection against a subsequent pathogenic infection. The protective effect of insect immune priming has been linked to the activation of humoral or cellular features of the innate immune response during the preliminary challenge, and these effects might last long enough to promote the survival of the infected animal. The fruit fly *Drosophila melanogaster* is a superb model to dissect immune priming processes in insects due to the availability of molecular and genetic tools, and the comprehensive understanding of the innate immune response in this organism. Previous investigations have indicated that the *D*. *melanogaster* immune system can be primed efficiently. Here we have extended these studies by examining the result of immune priming against two potent entomopathogenic bacteria, *Photorhabdus luminescens* and *P*. *asymbiotica*. We have found that rearing *D*. *melanogaster* on diet containing a non-pathogenic strain of *Escherichia coli* alone or in combination with *Micrococcus luteus* upregulates the antibacterial peptide immune response in young adult flies, but it does not prolong fly life span. Also, subsequent intrathoracic injection with *P*. *luminescens* or *P*. *asymbiotica* triggers the Immune deficiency and Toll signaling pathways in flies previously exposed to a live or heat-killed mix of the non-pathogenic bacteria, but the immune activation fails to promote fly survival against the pathogens. These findings suggest that immune priming in *D*. *melanogaster*, and probably in other insects, is determined by the type of microbes involved as well as the mode of microbial exposure, and possibly requires a comprehensive and precise alteration of immune signaling and function to provide efficient protection against pathogenic infection.

## Introduction

The previous exposure of an insect host to live or dead microbes can induce immune priming, a process that can promote activation of certain innate immune activities, which can in turn provide a protective effect against a subsequent infection [1]. Previous evidence suggests the existence of various forms of immune priming effects in different insect species. For instance, *Manduca sexta* larvae pre-injected with live bacteria of a non-pathogenic strain of *E*. *coli* are better protected to a subsequent injection with the insect-specific pathogen *Photorhabdus luminescens* due to substantial upregulation of the antimicrobial peptide response [2]. Feeding larvae of the cabbage looper (*Trichoplusia ni*) on a diet containing or lacking non-pathogenic *E*. *coli* and *M*. *luteus* bacteria has led to substantial upregulation of several genes encoding antimicrobial peptides, storage proteins, recognition proteins and melanisation related enzymes in the midgut, suggesting that the presence of bacteria in the gut lumen can not only be detected but also induce a response [3]. Using a similar experimental setup, honey bee (*Apis mellifera*) larvae exposed through feeding to spores of *Paenibacillus larvae* or to a mix of non-pathogenic bacteria have also exhibited increased upregulation of the antimicrobial peptide gene *abaecin* [4]. Also, mRNA levels of *cecropin A* are notably elevated in the fat body, gut and hemocytes of the silkworm (*Bombyx mori*) previously fed on diet supplemented with heat-killed *P*. *aeruginosa* cells compared to untreated controls [5]. Furthermore, *Bombus terrestris* bumblebees have increased survival to injection with *Pseudomonas fluorescens*, *Paenibacillus alvei*, or *P*. *larvae* following pre-injection the same pathogenic bacterium [6]. These findings indicate that immune priming in insects can regulate molecular processes controlling specific immune functions that could then impact the outcome of an infection.

In the *D*. *melanogaster* model, flies injected with live or dead cells of the Gram-positive bacterium *S*. *pneumoniae* were able to survive and eliminate a secondary injection with this pathogen. This proved to be a bacterium-specific effect, which was due to increased activation of the Toll pathway and enhanced phagocytic capacity in the primed flies [7]. Similarly, *D*. *melanogaster* flies exhibited prolonged survival in response to injection with a virulent strain of *Pseudomonas aeruginosa* following pre-injection with a non-virulent strain of this bacterium. Although in this case the effect was short-lasting, again it was attributed to changes in humoral and cellular immune activities in the pre-infected insects [8]. Interestingly, pre-exposure of adult flies through pricking to *Drosophila* C Virus (DCV) does not improve their survival to a subsequent infection by pricking with this natural viral pathogen [9]. These findings indicate that priming efficiency in *D*. *melanogaster* can be variable depending on the type of microbes and mode of experimental infection.

Bacteria from the genus *Photorhabdus* can act as mutualists and pathogens in different hosts [10]. *P*. *luminescens* are bioluminescent Gram-negative bacilli (Enterobacteriaceae), which live together with the entomopathogenic nematodes *Heterorhabditis bacteriophora* and establish pathogenic infections in insects [11–13]. The bacteria are vectored in the gut of the infective juvenile stage of the nematodes, which live in the soil and frequently invade susceptible insect larvae. The bacteria are released once the nematodes enter the hemolymph (insect blood), where they multiply rapidly and simultaneously produce a diverse range of toxins and metabolites that target vital insect tissues and organs, causing apoptosis of mainly the gut and fat body cells as well as hemocytes that patrol the hemocoel (insect body cavity) [14]. The high virulence of *P*. *luminescens* is also attributed to the ability of these bacteria to interfere with innate immune reactions of the colonized insect through the production of cytotoxic molecules that repress phenoloxidase activity and the melanization cascade, hemocyte spreading, aggregation, nodule formation, phagocytosis and microbial encapsulation, and the antimicrobial peptide response [15]. The related species *P*. *asymbiotica* is also highly virulent to insects but also an opportunistic human pathogen [16,17]. Their pathogenic properties are controlled by molecular regulatory systems that promote bacterial survival and pathogenicity towards insects by preventing hemocyte migration and phagocytosis [18,19].

In the current study, we aimed at examining the priming effect of *D*. *melanogaster* larvae having non-pathogenic bacteria in their environment and subsequently infecting the emerged flies with the insect pathogens *P*. *luminescens* or *P*. *asymbiotica*. In particular, our goal was to determine whether pre-exposure of *D*. *melanogaster* to non-pathogenic bacteria affects antimicrobial peptide gene expression and whether immune signaling activation due to immune priming or to a subsequent pathogenic infection provides a survival advantage to the adult flies.

## Results

### Pre-exposure of *Drosophila melanogaster* to non-pathogenic bacteria during development does not increase antimicrobial peptide gene expression and longevity

**Fig 1:**
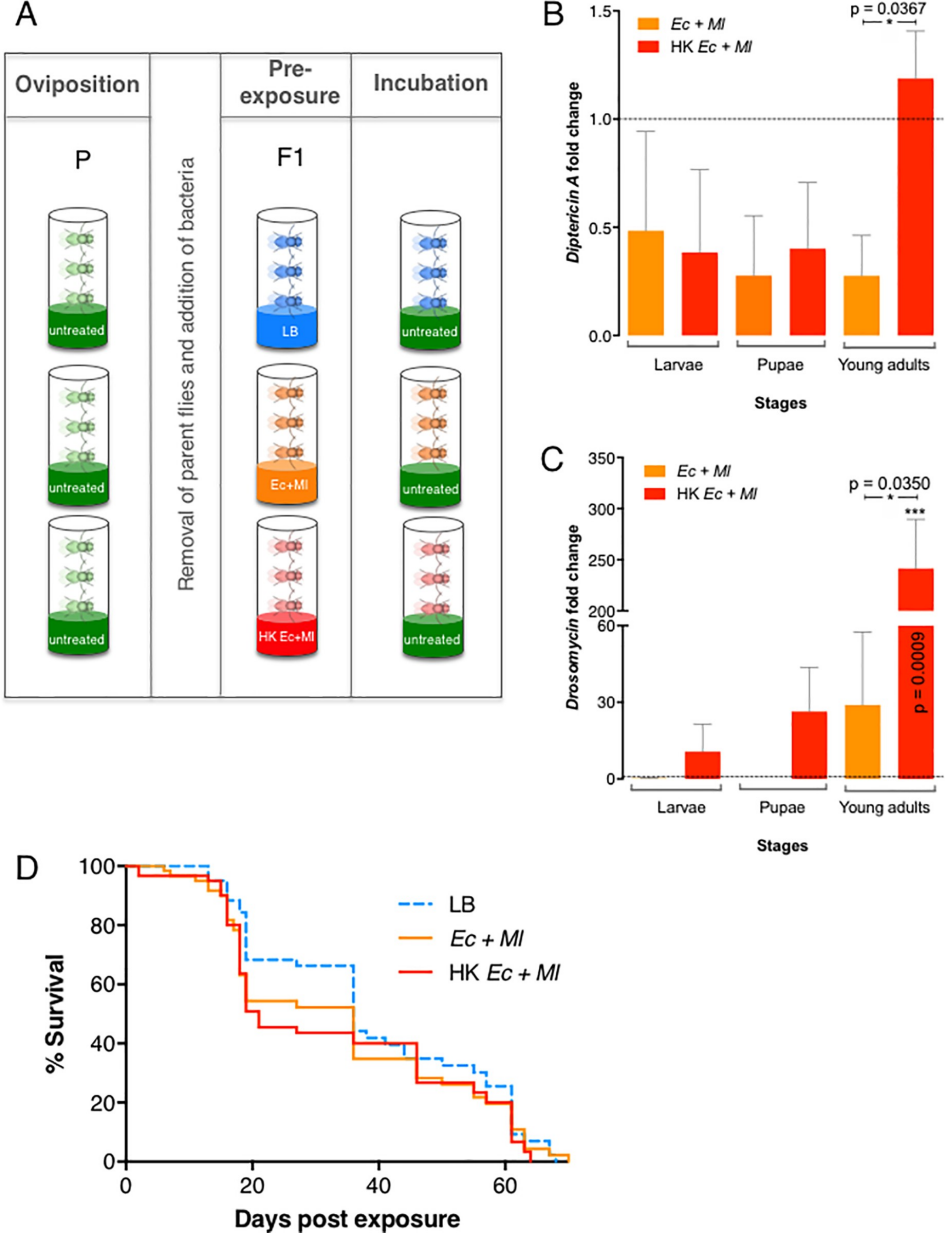
*Drosophila melanogaster* bacterial pre-exposure. (A) Experimental scheme. *Oregon-R* wild-type flies (P, Parental generation) were reared and allowed to oviposit on normal untreated diet. After parent removal, the progeny (F1 generation) completed their development on diet supplemented with Luria-Bertani media (LB), a mix of live *Escherichia coli* and *Micrococcus luteus* (Ec+Ml), or a mix of heat-killed bacteria (HK Ec+Ml) (Pre-exposure). (B) Effect of pre-exposure to live (Ec+Ml) or dead (HK Ec+Ml) bacteria on the transcript levels of the antimicrobial peptide genes *Diptericin-A* (B) and *Drosomycin* (C) in larvae, pupae and young adult flies (1–3 day-old) of *D*. *melanogaster*. All values were normalized to LB-containing media controls and analyzed using unpaired t-test. Error bars represent standard error of the mean. (D) Longevity of *D*. *melanogaster* following pre-exposure to live (Ec+Ml) or dead (HK Ec+Ml) bacteria. Fly mortality for each treatment is shown over a 70-day period. A log-rank (Mantel Cox) test performed using GraphPad Prism software did not detect significant differences between the survival curves (P = 0.3873), indicating that any effect on longevity produced by pre-exposure is relatively slight and inconsequential.

In order to identify the effect of bacterial pre-exposure on *D*. *melanogaster* immune activation, eggs hatched in the presence or absence of Gram-negative and Gram-positive bacteria (Fig 1A). Specifically, emerged larvae were reared in the presence of live *E*. *coli* and *M*. *luteus*, a heat-killed mix of both bacteria, or liquid media alone that served as control treatment. Analysis of antimicrobial peptide gene expression showed that *Diptericin A* and *Drosomycin* transcript levels were not significantly altered in larvae and pupae, but they were significantly increased in young adult flies (1–3 day-old) that had been reared in contact with live or heat-killed bacteria compared to non-pre-exposed individuals (Fig 1B and 1C; and S1 and S2 Tables).

We then monitored the life span of *D*. *melanogaster* for a period of 70 days, at which point all flies had died. We found no significant differences in longevity among flies that had been subjected to various pre-exposure treatments (P = 0.3873; Fig 1D and S4 Table). During the period between approximately 20 and 40 days, survival curves appeared to separate in a way that could indicate a pattern, but the slight trend proved unsustainable, and was not substantial enough to be attributed to treatment conditions.

### Antimicrobial peptide gene expression increases in *Drosophila melanogaster* pre-exposed to non-pathogenic bacteria and subsequently infected with *Photorhabdus* pathogenic bacteria

**Fig 2:**
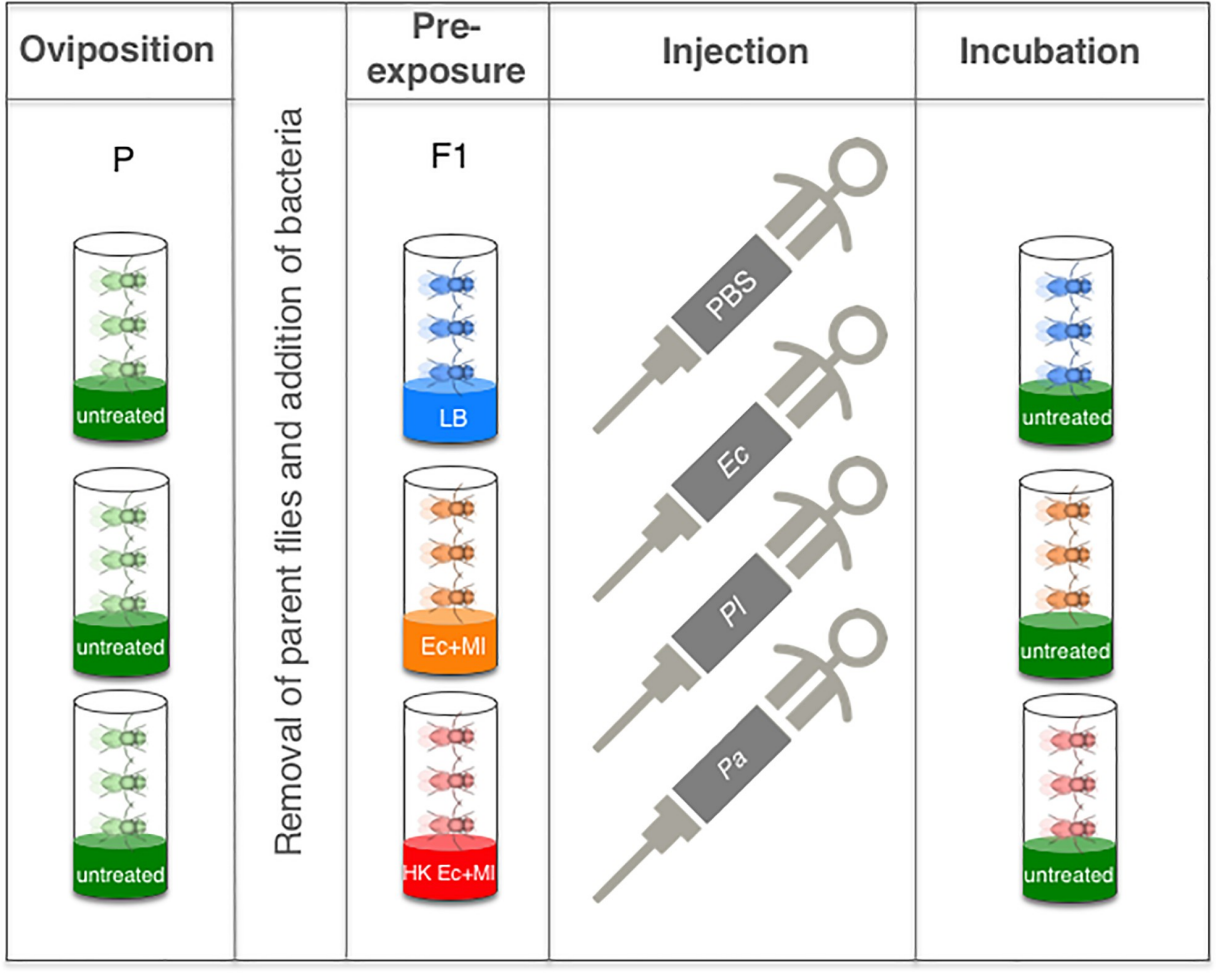
Experimental set up for *Drosophila melanogaster* bacterial pre-exposure, subsequent infection, and incubation. *Oregon-R* wild-type flies (P, Parental generation) were raised and allowed to lay eggs on normal untreated food. Parent flies were then removed from the vials and the progeny (F1 generation) were fed on food containing a mix of live *Escherichia coli* and *Micrococcus luteus* (Ec+Ml), a mix of heat-killed bacteria (HK Ec+Ml) or on control media supplemented with Luria-Bertani (LB) (Pre-exposure). Pre-exposed flies were then injected intrathoracically with phosphate-buffered saline (PBS), a non-pathogenic strain of *E*. *coli*, or the insect pathogenic bacteria *Photorhabdus luminescens* and *P*. *asymbiotica*. After injection, adult flies were incubated in vials containing normal untreated media.

**Fig 3:**
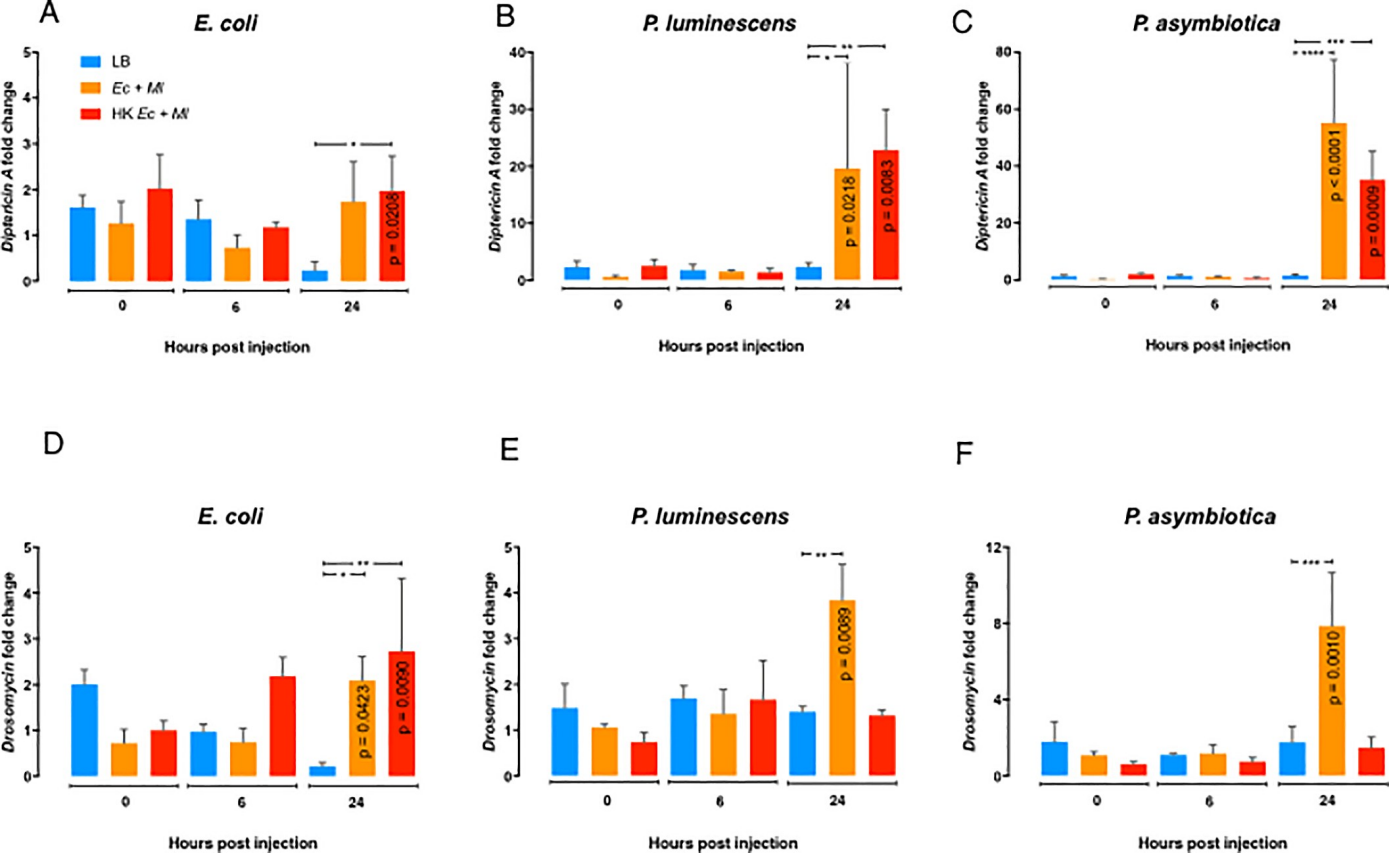
Antimicrobial peptide gene expression in *Drosophila melanogaster* pre-exposed to non-pathogenic bacteria and subsequently injected with pathogenic bacteria. Transcript levels of *Diptericin A* (A-C) and *Drosomycin* (D-F) at 0, 6, and 24 hours following intrathoracic injection with phosphate-buffered saline (PBS), *Escherichia coli*, *Photorhabdus luminescens*, or *P*. *asymbiotica*. Prior to injection, *D*. *melanogaster* flies were grown on fly food mixed with Luria-Bertani (LB), *E*. *coli* and *M*. *luteus* mix (*Ec+Ml*), or heat-killed *E*. *coli* and *M*. *luteus* mix (HK *Ec+Ml*). All values were normalized to the PBS-injected control flies at the corresponding time points and analyzed using two-way ANOVA. Error bars represent standard error of the mean (*P≤0.05, ***P≤0.001, ****P ≤0.0001).

Following bacterial pre-exposure, emerged adult flies were aged for 5 to 7 days and then injected with PBS (negative control), *E*. *coli* (non-pathogenic control), or the entomopathogenic bacteria *P*. *luminescens* or *P*. *asymbiotica* (Fig 2). To monitor immune signalling activation in *D*. *melanogaster* flies subjected to bacterial pre-exposure against the *Photorhabdus* pathogens, we measured the transcript levels of the Immune deficiency (Imd) and Toll pathway regulated antimicrobial peptide encoding genes *Diptericin A* and *Drosomycin*, respectively, using qRT-PCR and gene-specific primers (Fig 3 and S3 Table). We observed higher transcript levels of *Diptericin A* and *Drosomycin* at 24 h post injection with *E*. *coli* in flies pre-exposed to live or dead *E*. *coli* and *M*. *luteus* bacteria compared to flies treated with LB (Fig 3A and 3D). Similarly, we found that pre-exposure with live or heat-killed bacteria induced transcript levels of *Diptericin A* at 24 h post injection with *P*. *luminescens* or *P*. *asymbiotica* (Fig 3B and 3C), whereas *Drosomycin* was upregulated at the same time point in response to either *Photorhabdus* species injected into flies that had been previously exposed to a mix of live, but not to heat-killed, non-pathogenic bacteria (Fig 3E and 3F).

### Pre-exposure of *Drosophila melanogaster* to non-pathogenic bacteria during development does not protect against subsequent infection with *Photorhabdus*

**Fig 4:**
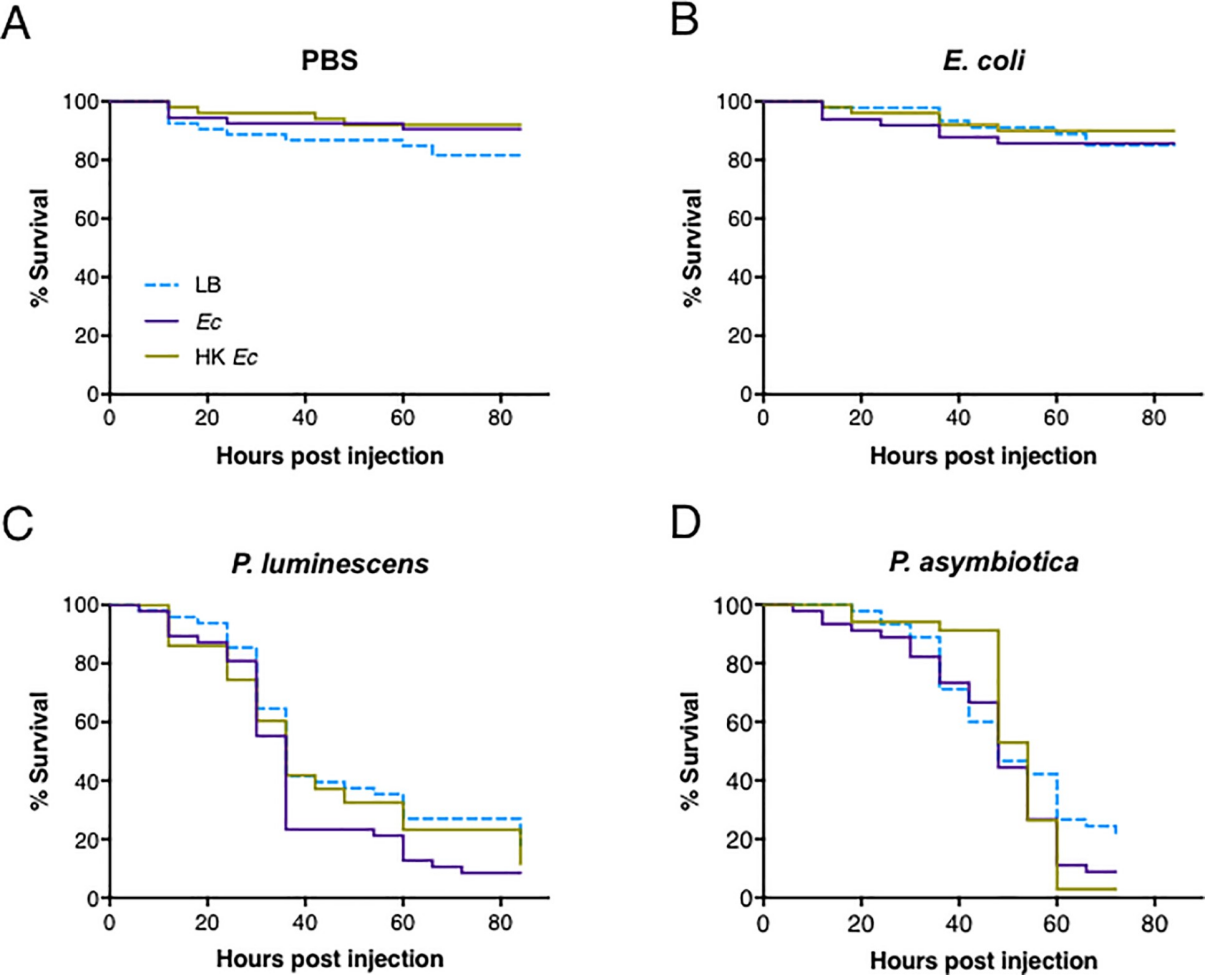
Survival of *Drosophila melanogaster* flies pre-exposed to non-pathogenic bacteria and subsequently injected with pathogenic bacteria. Emerged flies pre-exposed to Luria-Bertani (LB) broth, a non-pathogenic strain of *Escherichia coli* (Ec), or heat-killed *E*. *coli* (HK Ec) were transferred to fresh untreated diet and then injected with (A) phosphate-buffered saline (PBS), (B) *E*. *coli*, (C) *Photorhabdus luminescens*, or (D) *P*. *asymbiotica*. Survival was monitored for 72 hours post injection. Survival experiments were replicated three times and results were analyzed using Log-rank (Mantel-Cox) test in GraphPad Prism software.

**Fig 5:**
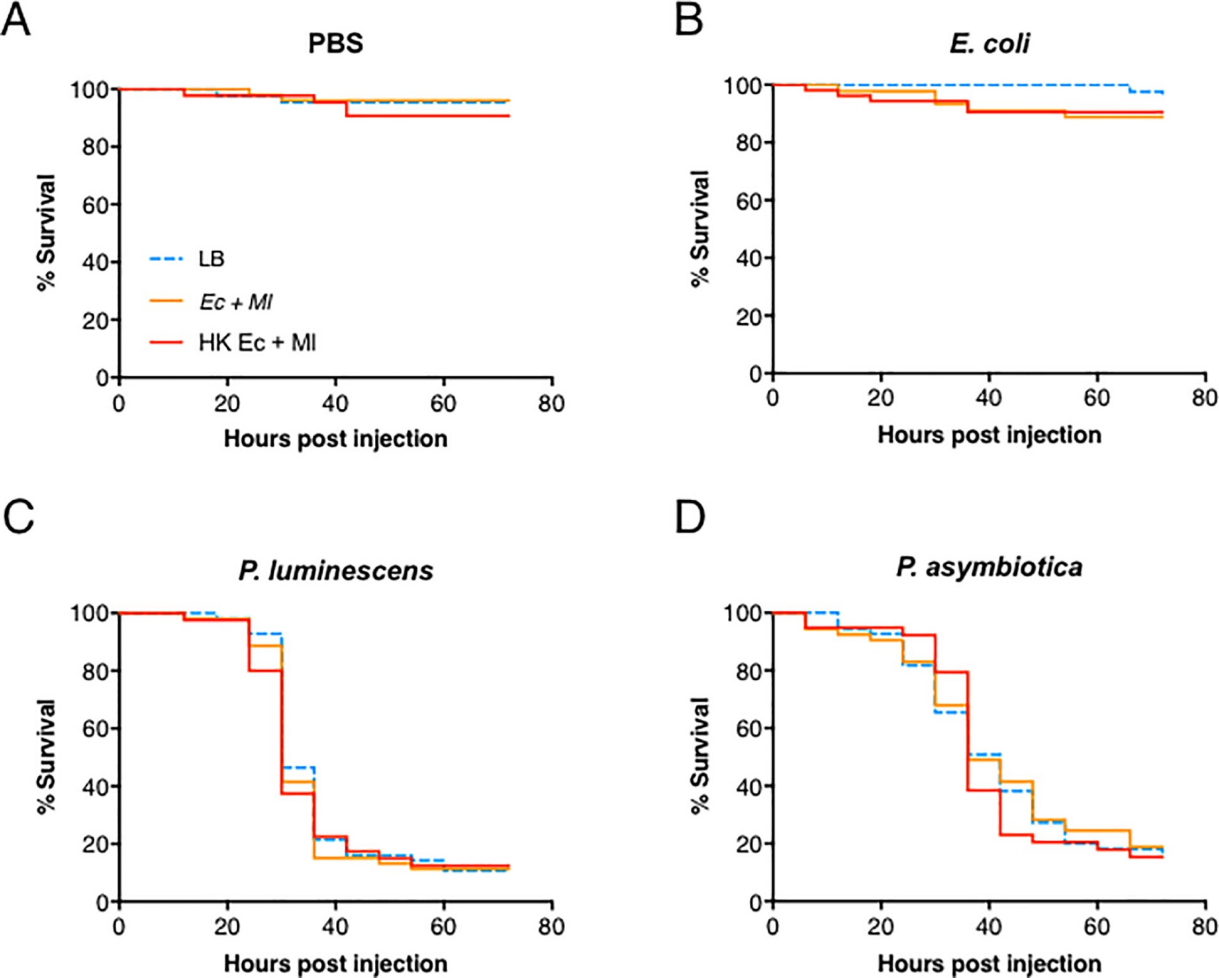
Survival of *Drosophila melanogaster* flies pre-exposed to a non-pathogenic bacterial mix and subsequently injected with pathogenic bacteria. Emerged flies pre-exposed to Luria-Bertani (LB) broth, a mix of live *Escherichia coli* and *Micrococcus luteus* (Ec+Ml), or a mix of heat-killed *E*. *coli* and *M*. *luteus* (HK Ec+Ml) were transferred to fresh untreated diet and then injected with (A) phosphate-buffered saline (PBS), (B) *E*. *coli*, (C) *Photorhabdus luminescens*, or (D) *P*. *asymbiotica*. Survival was monitored for 72 hours post injection. Survival experiments were replicated three times and results were analyzed using Log-rank (Mantel-Cox) test in GraphPad Prism software.

Based on previous evidence [1,6,20], we reasoned that wild-type flies pre-exposed to non-pathogenic bacteria during development would potentially survive differently in response to subsequent infection with pathogenic or non-pathogenic bacteria. Flies were raised in different conditions that included food supplemented with LB medium, and food containing either live or heat-killed *E*. *coli* bacteria. We monitored the survival of pre-exposed *Oregon-R* flies following injection with pathogenic *P*. *luminescens* or *P*. *asymbiotica* bacteria. Flies pre-exposed to either live or heat-killed *E*. *coli* did not die after injection with PBS or *E*. *coli* (Fig 4A and 4B, respectively; S4 Table). Also, flies pre-exposed to live or heat-killed *E*. *coli* were similarly sensitive to injection with either *P*. *luminescens* or *P*. *asymbiotica* compared to control flies reared on food without bacteria (Fig 4C and 4D, respectively). Flies pre-exposed to either live or heat-killed mix of *E*. *coli* and *M*. *luteus* did not die in response to PBS or *E*. *coli* injection (Fig 5A and 5B; S4 Table), and they were as sensitive as control LB treated flies following injection with either *Photorhabdus* pathogen (Fig 5C and 5D).

## Discussion

Results from previous studies have indicated that pre-exposure of *D*. *melanogaster* or other insects to non-pathogenic bacteria or a low sub-lethal dose of certain pathogenic bacteria can promote the survival response to a succeeding infection with pathogenic microbes. This effect has been attributed mainly to pre-activation of humoral aspects of the insect immune system [1,20–23]. Here, we have analyzed the consequences of pre-exposing *D*. *melanogaster* to non-pathogenic Gram-negative and Gram-positive bacteria on the survival ability and activation of immune signaling in response to the potent entomopathogenic bacteria *P*. *luminescens* and *P*. *asymbiotica*. We have found that pre-exposure during development of *D*. *melanogaster* with live or heat-killed bacteria increases the antimicrobial peptide gene expression in young adult flies but fails to prolong fly longevity. The pre-exposure effect can further trigger the expression of antimicrobial peptide genes upon secondary infection through intrathoracic injection with the entomopathogen *Photorhabdus*, which is what would be expected from priming [20]. However, the observed immune activation fails to confer a survival advantage to the bacterially pre-exposed flies when subsequently infected with either species of *Photorhabdus*.

Our current results denote that maintaining *D*. *melanogaster* in contact with live or dead mix of non-pathogenic Gram-negative and Gram-positive bacteria induces the Toll pathway at higher levels than the Imd pathway. However, injection of live or dead *E*. *coli* or *M*. *luteus* cells into wild-type adult flies has been shown previously to cause stronger upregulation of Imd and Toll pathway read-out genes, respectively [24,25]. These results suggest that induction of certain immune signaling pathways in *D*. *melanogaster* responding to non-pathogenic bacteria can vary greatly according to the mode of delivery as well as the developmental stage of the host. Therefore, given that the Imd pathway is mainly induced in the fat body and gut of *D*. *melanogaster* [26], we suspect that the low transcript levels of *Diptericin A* in pre-exposed larvae and young adult flies is probably due to the fewer numbers of bacterial cells that would normally gain access to the gut tissue compared to pre-infection through direct injection of bacterial cells into the hemolymph of *Drosophila* adult flies.

Our results also reveal that raising *D*. *melanogaster* in the presence of live or dead mix of *E*. *coli* and *M*. *luteus* upregulates Imd and Toll signaling in the pre-exposed flies subsequently injected with *Photorhabdus* pathogens. These findings suggest that although Imd and Toll pathways are not substantially induced in larvae and pupae, but only in young adult flies, being in contact with non-pathogenic bacteria, the activity of both pathways can be enhanced substantially in flies experiencing a secondary infection with pathogenic or non-pathogenic bacteria, such as *E*. *coli* and the two *Photorhabdus* species. The late upregulation of Imd signaling in response to *Photorhabdus* injection might be due to the activation of the immune receptor Persephone which is able to sense a broad range of microbes through virulence factor activities rather than molecular patterns [27]. Similar to our results, larvae of the mosquito *Aedes aegypti* fed on *E*. *coli* have failed to show any signs of upregulation in antibacterial peptide gene transcript levels; however, *attacin* and *defensin* were upregulated only upon subsequent injection with these bacteria [28]. Of note, reduced antimicrobial peptide gene expression in insects pre-exposed to non-pathogenic bacteria compared to untreated controls has not been shown so far. Overall, previous and current findings indicate that priming of the insect antibacterial immune response can vary greatly according to the insect species, the specific tissues examined, the type of microbe used as priming agent, and the type of pathogen used for secondary challenge.

We have found that longevity is not affected when rearing *D*. *melanogaster* in the presence of *E*. *coli* and *M*. *luteus*, and although Imd and Toll signaling are upregulated after injection of *P*. *asymbiotica* in flies that had been pre-exposed to the non-pathogenic bacteria, the survival response of the primed flies to *Photorhabdus* remains unaffected. These findings signify that induction of the antibacterial peptide response in primed *D*. *melanogaster* is not sufficient to enhance or diminish the survival ability of the flies to this potent entomopathogen. In line with the current results, Imd and Toll signaling can be activated in response to *P*. *luminescens* infection; however, Imd and Toll deficient mutants fail to show increased sensitivity to this pathogen [29]. Previous studies have further reported that pre-injection or oral infection of certain insect species with non-pathogenic bacteria can either confer a protective effect or provide no substantial survival advantage to secondary pathogenic infection. For instance, pricking of *D*. *melanogaster* flies with a low dose of DCV has proven insufficient to protect against subsequent pricking with a lethal dose of this virus, which could be due to short-lasting immune effect of the primary challenge on preventing or suppressing the lethal activity of the secondary viral infection [9]. Similarly, injection of pea aphids *Acyrthosiphon pisum* to a heat-killed mix of *E*. *coli* and *M*. *luteus* provides no protection to secondary challenge through injection with live pathogenic *Serratia marcescens* or non-pathogenic *E*. *coli*, suggesting lack of antibacterial priming effect in this aphid species [30]. In contrast, oral exposure of red flour beetle (*Tribolium castaneum*) larvae to liquid media previously used for growing *Bacillus thuringiensis* spores confers a bacterial strain-specific priming effect by promoting survival to oral infection with this insect pathogen [31]. A bacterial species-specific survival protective effect has also been documented for the tiger moth *Parasemia plantaginis* when larvae are orally exposed to a non-lethal dose of the pathogenic bacteria *S*. *marcescens*, but not to non-pathogenic *E*. *coli*, and later injected with a high number of *S*. *marcescens* cells [32]. In addition, *B*. *mori* larvae fed on diet containing dead *P*. *aeruginosa* cells display increased survival ability towards subsequent injection with these bacteria, which is accompanied by lower pathogen replication and higher levels of antimicrobial peptide activity in the hemolymph of the primed insects [5].

In conclusion, in the present study we have shown that the constant presence of non-pathogenic bacteria during the development of *D*. *melanogaster* can lead to systemic activation of antimicrobial peptide gene expression but fails to provide protection to subsequent infection with the potent insect pathogen *Photorhabdus* or non-pathogenic *E*. *coli*, despite the induction of immune signaling upon challenge with these bacteria. These findings, in combination with previous reports [9,30], support the notion that priming of *D*. *melanogaster* through microbial pre-exposure does not always provide a consistent positive impact on survival to a secondary pathogenic challenge. Identifying the exact basis for variation in immune priming capacity of *D*. *melanogaster* as well as in other insects will require the employment and efficient application of systems biology approaches, which will allow the precise dissection of the molecular and physiological processes and their distinct components that strengthen or impede innate immune activity against a range of natural and non-natural pathogenic microorganisms.

## Materials and methods

### Fly stocks

Wild-type *Oregon-R* flies were raised on standard cornmeal-soy based food (Cat. No. 101-NV, Meidi laboratories) supplemented with a few granules of dry baker’s yeast at 25°C, 12:12 light/dark photoperiod, and 60% humidity.

### Bacterial stocks

Bacterial strains *Escherichia coli* K12, *Micrococcus luteus* (CIP A270), *P*. *luminescens* TT01 and *P*. *asymbiotica* ATCC43943 were plated from glycerol stocks on Luria-Bertani (LB) agar plates and kept overnight at 37°C for *E*. *coli* and 30°C for *M*. *luteus* and the *Photorhabdus* strains. Single colonies were inoculated in 10 ml of LB media and incubated overnight at 37°C (*E*. *coli*) or 30°C (*M*. *luteus*, *P*. *luminescens* and *P*. *asymbiotica*) and constant agitation (210 rpm). Cultures were centrifuged at 2935x g for 10 minutes at 4°C. Supernatants were discarded and pellets were washed with 10 ml of sterile 1X phosphate buffered saline (PBS) (Sigma). For treatments with live bacteria, *E*. *coli* (3.8x10^9^ cells/ml) or mixture of *E*. *coli* and *M*. *luteus* (3.8x10^9^ and 4.4x10^7^ cells/ml) pellets from overnight cultures were re-suspended in 10 ml of LB and 300 μl of the suspension were added to 10 ml of food in the fly vials. For treatments with heat-killed bacteria, *E*. *coli* and *M*. *luteus* pellets from overnight cultures were added to 10 ml of sterile 1X PBS, heated at 95°C for 30 minutes and 300 μl of the heat-killed bacteria were re-suspended in LB and then added to the fly vials.

### Bacterial pre-exposure setup

Three replicates of five male and female adult flies were initially reared on normal fly food, as described in Fig 1A, P(arental) panel. After two days of egg laying, parent adult flies were removed, leaving only progeny (F1) in the vials. Food was supplemented with 300 μl of a mixture of live *E*. *coli* and *M*. *luteus* or a mixture of heat-killed bacteria. Control treatments involved the addition of 300 μl of LB media to the vials.

### Longevity tests

Five male and five female newly emerged flies (Fig 1, F1) from each treatment condition were placed in vials containing untreated food. The flies were kept at 25°C and surviving individuals were transferred to new vials every five days to prevent death due to the waterier food characteristic of a growing larval population. Fly mortality was assessed every 24 hours until mortality for each treatment reached one hundred percent. Longevity curves were generated from three independent trials.

### Bacterial injections

For injections, bacterial strains were grown as described above and bacterial pellets were finally re-suspended in 6 ml of sterile 1X PBS, 20 μl of *E*. *coli*, 150 μl of *P*. *luminescens* and 300 μl of *P*. *asymbiotica*. Bacterial concentrations for each suspension were adjusted to optical density (600 nm) of 0.015 for *E*. *coli*, 0.1 for *P*. *luminescens*, and 0.25 for *P*. *asymbiotica* (which correspond to 100–300 colony forming units per fly of each bacterial preparation) using a spectrophotometer (NanoDrop 2000c; Thermo Fisher Scientific).

### Survival experiments

After pre-exposure to live or heat-killed *E*. *coli* alone or mixed with *M*. *luteus* bacteria, emerged flies were collected and aged for injections (Fig 2). Adult flies (5–7 day-old) were challenged with *E*. *coli*, *P*. *luminescens* or *P*. *asymbiotica* by intrathoracic injection (18.4 nl) using Nanoject II (Drummond Scientific). Flies were injected with the same amount of PBS as a septic injury control. All injected flies were subsequently kept in groups of 10 (five males and five females) at 25°C on untreated media. Fly survival was monitored every six hours and up to 72 hours following injection. Three independent survival experiments were performed.

### Gene expression assays

During the bacterial pre-exposure period, *D*. *melanogaster* 4–6 third instar larvae, pupae, and young adult flies (1–3 day-old) were collected for gene expression assays. Following bacterial pre-exposure, a mix of five male and five female adult flies (5–7 day-old) were injected with 18.4 nl of PBS, *E*. *coli*, *P*. *luminescens*, or *P*. *asymbiotica* and frozen at 0, 6, and 24 hours. Total RNA was extracted by adding 500 μl of TRIzol (Invitrogen, Carlsbad, CA) into pooled homogenized flies, followed by 100 μl of 1-bromo-3-chloropropane (Acros Organics). After centrifugation at 12000 *g* for 15 minutes at 4°C, the top clear phase was transferred to a fresh tube containing 1 μl of glycogen (VWR), mixed with 500 μl of isopropanol (Fisher Scientific), and incubated for 15 minutes at room temperature. After another round of centrifugation and supernatant removal, the pellet was washed with 1 ml of 75% ethanol (Decon Labs), air-dried and then dissolved in 20 μl of autoclaved deionized water before heated up at 56°C for 10 minutes. RNA concentration was determined using Nanodrop 2000C (Thermo Scientific). cDNA was synthesized from 500 ng of extracted total RNA using High-Capacity cDNA Reverse Transcription Kit (Applied Biosystems) with addition of Murine RNase Inhibitor (New England Biolabs). The resulting cDNA was consequently diluted 5X in autoclaved deionized water. iTaq Univer SYBR Green (Bio-Rad) was used for quantitative real-time polymerase chain reaction (qRT-PCR) analysis using CFX96 Real-Time System, C1000 Thermal Cycler and the following conditions: 95°C for 2 min, 40 cycles of 95°C for 15 sec and 61°C for 30 sec, 95°C for 15 sec, 65°C for 5 sec and 95°C for 5 sec. Primer sets included RpL32FW 5´-GATGACCATCCGCCCAGCA-3´, RpL32RV 5´-CGGACCGACAGCTGCTTGGC-3´ (*RpL32*); DptFW 5´-GCTGCGCAATCGCTTCTACT-3´, DptRV 5´-TGGTGGAGTTGGGCTTCATG-3´ (*Diptericin A*); DrsFW 5´-GACTTGTTCGCCCTCTTCG-3´, DrsRV 5´-CTTGCACACACGACGACAG-3´ (*Drosomycin*) [33]. Three biological replicates were included per treatment and each biological replicate was analysed in technical duplicates.

### Statistical analyses

All data were plotted using Prism 7 (GraphPad Software). Statistical differences between survival curves and longevity curves were assessed using Log-rank (Mantel-Cox) test. Gene expression data were analyzed using the 2-^ΔΔCT^ method [34]. Analyzed data from pre-exposure experiments were compared using Unpaired t-test and injection experiments using one-way ANOVA (Fisher’s LSD).

## Supporting information

S1 Table Statistical analysis of gene expression of AMPs at different stages (Fig 1B and 1C) of *Drosophila* using one-way ANOVA with Fisher’s LSD comparing primed and non-primed individuals. (PDF)

S2 Table Statistical analysis of gene expression of AMPs at different stages (Fig 1B and 1C) of *Drosophila* using one-way ANOVA with Fisher’s LSD comparing live and heat-killed bacteria treatments. (PDF)

S3 Table Statistical analysis of gene expression of AMPs primed by non-pathogenic bacteria (Fig 3). (PDF)

S4 Table Statistical analysis of the survival graphs. (PDF)
