# Supplementary material for: Pre-exposure to non-pathogenic bacteria does not protect Drosophila against the entomopathogenic bacterium Photorhabdus
Source: PLoS One. 2018 Oct 31;13(10):e0205256. doi: 10.1371/journal.pone.0205256 (PMC6209181; doi:10.1371/journal.pone.0205256)
Supplement: S1 Table — (PDF) [file pone.0205256.s001.pdf]

S1 Table. Statistical analysis of gene expression of AMPs at different stages (Figs 1B and 1C) of *Drosophila* using one-way ANOVA with Fisher's LSD comparing primed and non-primed individuals.

Stages	Comparison	Individual P value	
		dpt-A	***drs***
Larvae	LB vs. Ec + Ml	0.2635	0.9916
	LB vs. HK Ec + Ml	0.1847	0.8993
Pupae	LB vs. Ec + Ml	0.1227	0.9896
	LB vs. HK Ec + Ml	0.1972	0.7413
Young Adult	LB vs. Ec + Ml	0.1223	0.7164
	LB vs. HK Ec + Ml	0.6248	0.0009
**ANOVA summary**			
F		2.06	6.163
P value		0.1097	0.0009
P value summary		ns	***
Significant diff. among means (P < 0.05)?		No	Yes
R square		0.4071	0.649

ns: not significant
