# Supplementary material for: Pre-exposure to non-pathogenic bacteria does not protect Drosophila against the entomopathogenic bacterium Photorhabdus
Source: PLoS One. 2018 Oct 31;13(10):e0205256. doi: 10.1371/journal.pone.0205256 (PMC6209181; doi:10.1371/journal.pone.0205256)
Supplement: S2 Table — (PDF) [file pone.0205256.s002.pdf]

S2 Table. Statistical analysis of gene expression of AMPs at different stages (Figs 1B and 1C) of *Drosophila* using one-way ANOVA with Fisher's LSD comparing live and heat-killed bacteria treatments.

Stages	Comparison	Individual P value	
		dpt-A	***drs***
Larvae	Ec + Ml vs HK Ec + Ml	0.8347	0.8967
Pupae		0.7946	0.7451
Young Adult		0.0368	0.0045
**ANOVA summary**			
F		1.979	6.126
P value		0.1369	0.0018
P value summary		ns	**
Significant diff. among means (P < 0.05)?		No	Yes
R square		0.3821	0.6299

ns: not significant
