# Supplementary material for: Pre-exposure to non-pathogenic bacteria does not protect Drosophila against the entomopathogenic bacterium Photorhabdus
Source: PLoS One. 2018 Oct 31;13(10):e0205256. doi: 10.1371/journal.pone.0205256 (PMC6209181; doi:10.1371/journal.pone.0205256)
Supplement: S3 Table — (PDF) [file pone.0205256.s003.pdf]

S3 Table. Statistical analysis of gene expression of AMPs primed by non-pathogenic bacteria (Fig 3).

Gene	Species	Hours	Comparison	P-value	ANOVA summary	
Diptericin-A	*Ec*	0	LB vs. *Ec + Ml*	0.6175		
			LB vs. HK *Ec + Ml*	0.5522	F	1.435
		6	LB vs. *Ec + Ml*	0.3707	P value	0.252
			LB vs. *HK Ec + Ml*	0.7986	P value summary	ns
		24	LB vs. *Ec + Ml*	0.0658	Significant diff. among means (P < 0.05)?	No
			LB vs. *HK Ec + Ml*	0.0208	R square	0.4031
	Pl	0	LB vs. *Ec + Ml*	0.7838		
			LB vs. HK *Ec + Ml*	0.9657	F	2.931
		6	LB vs. *Ec + Ml*	0.9689	P value	0.0347
			LB vs. *HK Ec + Ml*	0.9568	P value summary	*
		24	LB vs. *Ec + Ml*	0.0218	Significant diff. among means (P < 0.05)?	Yes
			LB vs. *HK Ec + Ml*	0.0083	R square	0.6099
	Pa	0	LB vs. *Ec + Ml*	0.8972		
			LB vs. HK *Ec + Ml*	0.9263	F	10.84
		6	LB vs. *Ec + Ml*	0.9705	P value	<0.0001
			LB vs. *HK Ec + Ml*	0.9098	P value summary	****
		24	LB vs. *Ec + Ml*	<0.0001	Significant diff. among means (P < 0.05)?	Yes
			LB vs. *HK Ec + Ml*	0.0009	R square	0.8525
Drosomycin	*Ec*	0	LB vs. *Ec + Ml*	0.1527		
			LB vs. HK *Ec + Ml*	0.2586	F	1.991
		6	LB vs. *Ec + Ml*	0.7945	P value	0.1073
			LB vs. *HK Ec + Ml*	0.1729	P value summary	ns
		24	LB vs. *Ec + Ml*	0.0423	Significant diff. among means (P < 0.05)?	No
			LB vs. *HK Ec + Ml*	0.009	R square	0.4695
	Pl	0	LB vs. *Ec + Ml*	0.5675		
			LB vs. HK *Ec + Ml*	0.3189	F	2.945
		6	LB vs. *Ec + Ml*	0.6849	P value	0.0341
			LB vs. *HK Ec + Ml*	0.9758	P value summary	*
		24	LB vs. *Ec + Ml*	0.0089	Significant diff. among means (P < 0.05)?	Yes
			LB vs. *HK Ec + Ml*	0.9249	R square	0.611
	Pa	0	LB vs. *Ec + Ml*	0.6532		
			LB vs. HK *Ec + Ml*	0.4563	F	4.317
		6	LB vs. *Ec + Ml*	0.9623	P value	0.0054
			LB vs. *HK Ec + Ml*	0.8202	P value summary	**
		24	LB vs. *Ec + Ml*	0.001	Significant diff. among means (P < 0.05)?	Yes
			LB vs. *HK Ec + Ml*	0.8682	R square	0.6701

ns: not significant
