# Supplementary material for: Pre-exposure to non-pathogenic bacteria does not protect Drosophila against the entomopathogenic bacterium Photorhabdus
Source: PLoS One. 2018 Oct 31;13(10):e0205256. doi: 10.1371/journal.pone.0205256 (PMC6209181; doi:10.1371/journal.pone.0205256)
Supplement: S4 Table — (PDF) [file pone.0205256.s004.pdf]

S4 Table. Statistical analysis of the survival graphs.

**Log-rank (Mantel-Cox) test**	**Fig 1D**	**Fig 4**				**Fig 5**			
		**PBS**	***Ec***	***Pl***	***Pa***	**PBS**	***Ec***	***Pl***	***Pa***
Chi square	3.028	2.3	0.4495	6.16	2.207	1.401	1.238	0.9581	0.2254
df	3	3	3	3	3	3	3	3	3
P value	0.3873	0.5126	0.9298	0.1041	0.5305	0.7054	0.7439	0.8114	0.9734
P value summary	ns	ns	ns	ns	ns	ns	ns	ns	ns
Are the survival curves sig different?	No	No	No	No	No	No	No	No	No

ns: not significant
